# Clinician-Focused Connected Health Requirements Gathering for Attention-Deficit/Hyperactivity Disorder Through Clinical Journey Mapping: Design Science Study

**DOI:** 10.2196/53617

**Published:** 2025-05-26

**Authors:** Richard Harris, Deirdre Murray, Angela McSweeney, Frederic Adam

**Affiliations:** 1 Department of Business Information Systems, Cork University Business School, University College Cork, 2.125 O’Rahilly Building, Cork, T12 K8AF, Ireland, 353212021110; 2 Department of Pediatrics and Child Health, College of Medicine & Health, University College Cork, Cork, Ireland; 3 Health Service Executive, Limerick, Ireland

**Keywords:** connected health, ADHD, Dundee clinical care pathway, integrated patient journey mapping tool, neurodevelopmental, design science, journey, map, mapping, diagram, visualization, attention deficit, information system, care pathway, design, implementation, integration, attention-deficit/hyperactivity disorder

## Abstract

**Background:**

Many health care systems globally face severe capacity issues, with lengthening waiting lists and stretched resources. Connected health has been proposed as a game changer for health care. However, the development of connected health apps is difficult and requires multidisciplinary development teams. Patient journey mapping presents an opportunity to streamline the requirements-gathering process for such apps by clearly showing the patient journey to team members who are not familiar with relevant clinical practices. This research project focuses on attention-deficit/hyperactivity disorder (ADHD) as a case study for using clinical journey mapping to represent the “gold standard” care pathway for ADHD treatment; the Dundee Clinical Care Pathway. This pathway was analyzed in detail and was further explored in discussions with stakeholders to produce a patient journey map.

**Objective:**

The objective of this paper is to answer three research questions: (1) visualizing the Dundee ADHD clinical care pathway using integrated patient journey mapping and exploring how its use benefits multidisciplinary development teams; (2) optimizing the integrated patient journey map arising from the Dundee Clinical Care Pathway, in line with the underlying clinical realities of Child and Adolescent Mental Health Service in Ireland; and (3) proposing areas where connected health integration can deliver efficiency and substantial gains for Child and Adolescent Mental Health Services.

**Methods:**

This study uses a design science approach where a sample artifact is presented to a relevant audience for review and feedback and is then leveraged to work iteratively toward an improved, final artifact. This paper presents the feedback collected from both information systems and clinical professionals at each iteration of the map.

**Results:**

This research delivers a comprehensive clinical patient journey map based on the Dundee clinical care pathway. Using unified modeling language concepts and color coding, multiple patient personas are mapped onto a streamlined diagram, allowing the diagram, at an abstract level, to cover the most typical clinical scenarios.

**Conclusions:**

Clinical journey mapping provides a way for team members to get up to speed on clinical practices, while also presenting a way for development teams to identify key gaps where connected health systems can be embedded in clinical pathways to optimize the use of clinical resources and ultimately deliver better patient outcomes.

## Introduction

Connected health is defined as *“*a conceptual model for health management where devices, services, or interventions are designed around the patient’s needs, and health-related–data is shared, in such a way that the patient can receive care in the most proactive and efficient manner possible*”* [1]. It incorporates telehealth and telemedicine, which describe situations where health care is provided without patients being necessarily face-to-face with the clinician, using technologies such as voice and video chat to provide services. Connected health systems have been shown to produce positive outcomes in terms of both treatment and participant satisfaction in many areas, including mental health services [2].

Ensuring that young patients have timely access to high-quality mental health services is critical, as adverse childhood experiences have a disproportionate impact when left untreated [3]. Yet, Child and Adolescent Mental Health Service (CAMHS) is struggling to meet demand. Reports indicate an increasing number of adolescents presenting to CAMHS for treatment. Figures from the United Kingdom show that the number of children with a diagnosed “mental disorder” is growing [4-7], with waiting lists for CAMHS running in months [8]. Connected health systems could play a key role in supporting CAMHS. As part of its action plan to modernize CAMHS in Ireland, the Maskey report [9] proposes to “explore the options available in Information and communication technology to improve the governance, effectiveness, efficiency, and accessibility of CAMHS,” namely by implementing electronic health records, electronic questionnaires, and generally, telemedicine on a broader scale [9]. This highlights the opportunity to embed connected health within CAMHS to achieve their digital transformation.

A critical step in ensuring the success of a connected health system is having a clear understanding of the experiences of patients during their treatment, the “pain points” they face, and where connected health systems can have an impact [10]. To capture the patient journey and the digitization opportunities within it, a visual representation is beneficial [11,12]. The “integrated patient journey mapping tool*”* (IPJM) [13], designed to support “multidisciplinary practitioners in designing health care solutions that meet the demands of existing constraints, performance improvement, and patient experience” [13,14] has been adopted in many studies. This paper builds on IPJM, using a design science approach to capture the Dundee Clinical Care Pathway [15], which is commonly used to manage attention-deficit/hyperactivity disorder (ADHD). ADHD is a common neurodevelopmental disorder, diagnosed in approximately 5% of children [16,17].

A key goal of this paper is, through patient journey mapping, to highlight areas where connected health systems could be embedded within the Dundee pathway. The Dundee pathway, originally developed in Scotland, creates a “clearly structured, evidence-based clinical pathway for the assessment and management of children and adolescents with ADHD” [15]. It has been identified in a systems review [9] as the preferred construct for the delivery of ADHD care and it is used in this paper as an initial template.

## Methods

### Research Questions

This paper proposes three research questions to effectively leverage the IPJM within the context of the Dundee pathway:

- Visualize the Dundee ADHD clinical care pathway using IPJM and explore how the use of IPJM benefits multidisciplinary development teams.
- Optimize the integrated patient journey map arising from the Dundee Clinical Care Pathway, in line with the underlying clinical realities of CAMHS in Ireland.
- Propose areas where connected health integration can deliver efficiency and substantial gains for CAMHS services.

The first research question justifies the use of IPJM to map the Dundee pathway, in the specific context of collaborative connected health systems development. The second question helps fine-tune the pathway, based on the accounts and experiences of key stakeholders toward improving its legibility [14], and the final research question looks to select specific steps and activities within the pathway where connected health can be leveraged to improve its efficiency.

### Design Science Process

**Figure 1.**
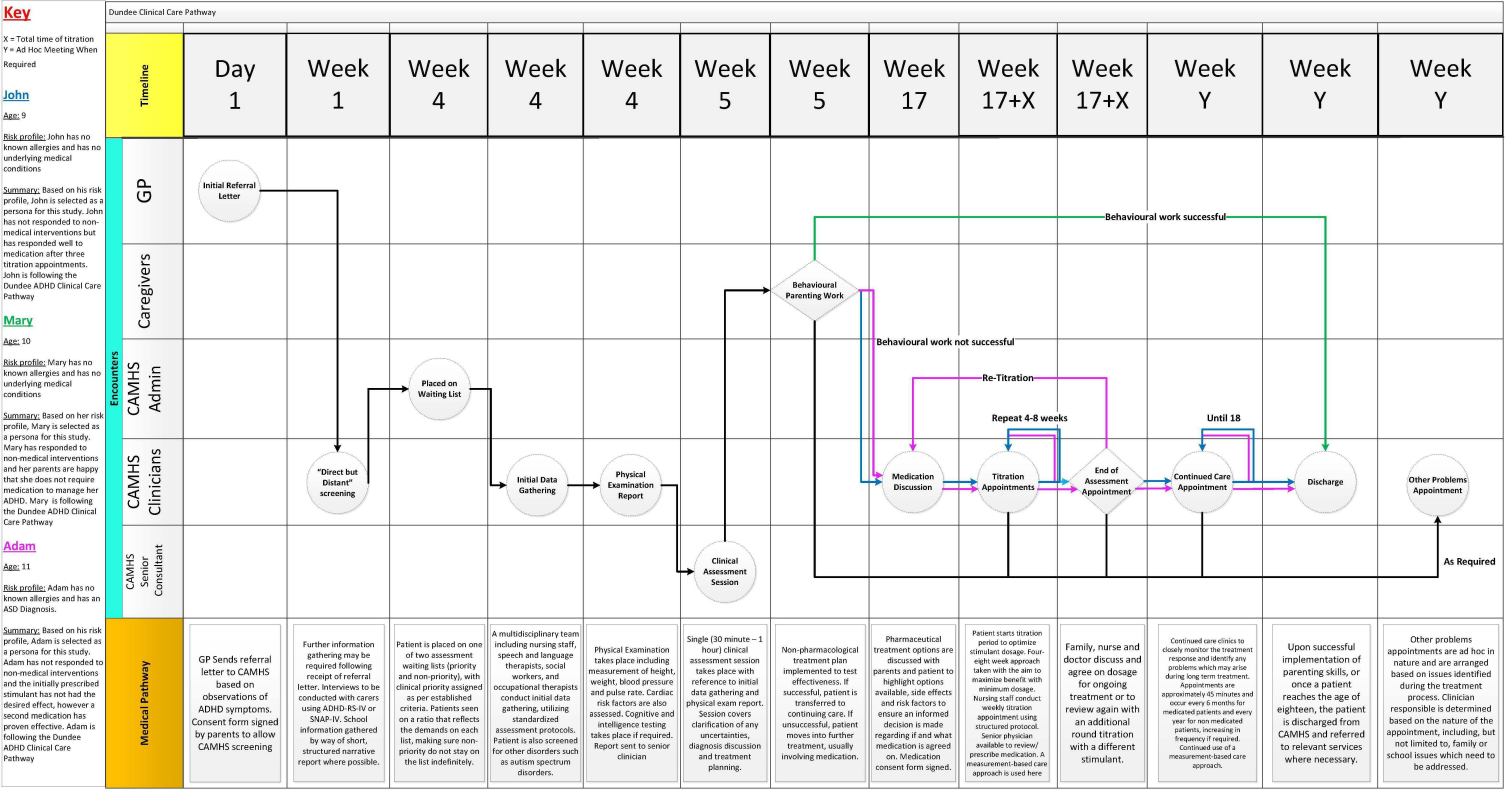
Third iteration of the PJMA, implementing feedback received from iteration two. Color coding changed to improve contrast and discharge point added, among other changes.

To populate the IPJM with personas of individuals based on the Dundee Clinical Care Pathway and to validate it, it is important to use a robust and rigorous approach that includes input from clinicians with various backgrounds, as well as academics from relevant disciplines, to ensure that the proposed visualization is correct and respects the guidelines of the model pathway. To achieve the desired result, a design science approach [18] was used to create and iterate through successive versions of the patient journey map artifact (PJMA). Design science is an approach involving “the creation of an artifact and/or design theory as a means to improve the current state of practice as well as existing research knowledge” [19]. Design science focuses on the development of a new artifact (eg, a diagram, app, or graph), as well as the study of the use of the newly created artifact. The research output for this project consists of this research paper which highlights the design of a diagram to visually illustrate the Dundee ADHD Clinical Care pathway, and the artefactual output consisting of the final diagram presented in Figure 1.

In rigorous application of the design science method, the design stages of the artifact, including clear illustrations of all iterations, with the feedback of the participants and the resulting alterations, are presented in detail. This ensures that the final design of the PJMA has been validated through a rigorous process and that the PJMA is a suitable representation of the Dundee ADHD Clinical Care Pathway.

Design science was chosen as the methodology of choice because (1) it is a generally accepted methodology within the information systems (IS) field that has proven to produce academically rigorous results, (2) it promotes an iterative process that permits feedback from various stakeholders to be used to refine the artifact design, similar to other research methodologies (eg, the Delphi study method [20]), and (3) it offers transparency in the development of the PJMA as each version is documented and presented as part of the final publication [21].

Interviews were conducted with individuals whose professional experience was relevant to the research area, these include information technology consultants, CAMHS clinicians, and IS researchers. Table 1 gives details of the professionals interviewed during the process. As the area of connected health within ADHD is a novel one, the use of qualitative data gathering and analysis in parallel with design science was viewed as a robust choice to build the ADHD case study, and subsequently, propose a novel theory [22].

**Table 1.** Total number of interviews by category.

Interview category	Number of interviews
CAMHS^a^ clinician	3
CAMHS nurse	1
Dundee pathway expert	1
Social worker	1
Connected health researcher	2
Connected health consultant	2
Total	10

a CAMHS: Child and Adolescent Mental Health Service.

**Figure 2.**
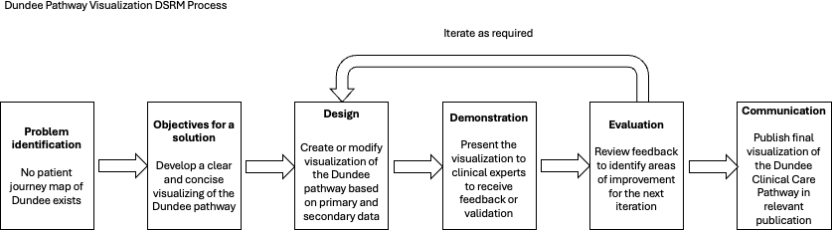
Design science research methodology, highlighting problem statement, solution objectives, and iterative development approach. DSRM: design science research methodology.

The results of each step within the design science research methodology [18] process for the development of the PJMA can be found in Figure 2, starting with the identification of the problem and objectives of the solution. Each iteration receives its own design and development, demonstration, and evaluation subsection, in line with design science research principles [18,19].

The objective of this research project is to create an accurate visual representation of the Dundee ADHD Clinical Care Pathway, which can be used as a visual aid for multidisciplinary teams who develop connected health apps to support clinicians in the treatment of ADHD. For the visual aid to be successful in conveying the Dundee pathway to viewers, it must do the following: (1) be true and accurate to the pathway as described by Coghill and Seth [15], (2) effectively convey the clinical journey through the pathway to individuals who have little to no clinical experience in treating or working with individuals diagnosed with ADHD, and (3) create an evaluation of IPJM using IS design principles to create a PJMA.

The three iterations conducted as part of this research project are presented in the following sections.

### Ethical Considerations

Approval was sought for this research project from University College Cork’s Social Research and Ethics Committee which approved both application Log 2022‐134 entitled “Connected Health Avenues for Adolescent Mental Health” on July 19, 2022, and subsequent amendment “2022‐134A” Informed consent was given by all participants prior to the commencement of the interviews. This was achieved by way of an information leaflet highlighting the study rationale, how findings would be used, and how the data would be stored and processed. Participants were then asked to sign a consent form to ensure they had read the information leaflet and were happy to participate. Due to ethical concerns, patients were not interviewed at this point of the project. CAMHS deals with adolescents and children who are receiving mental health care and therefore are classed as extremely vulnerable. This research has focused on mapping the pathway from a clinician’s view.

## Results

### Iteration One

#### Design and Development (Iteration One)

**Figure 3.**
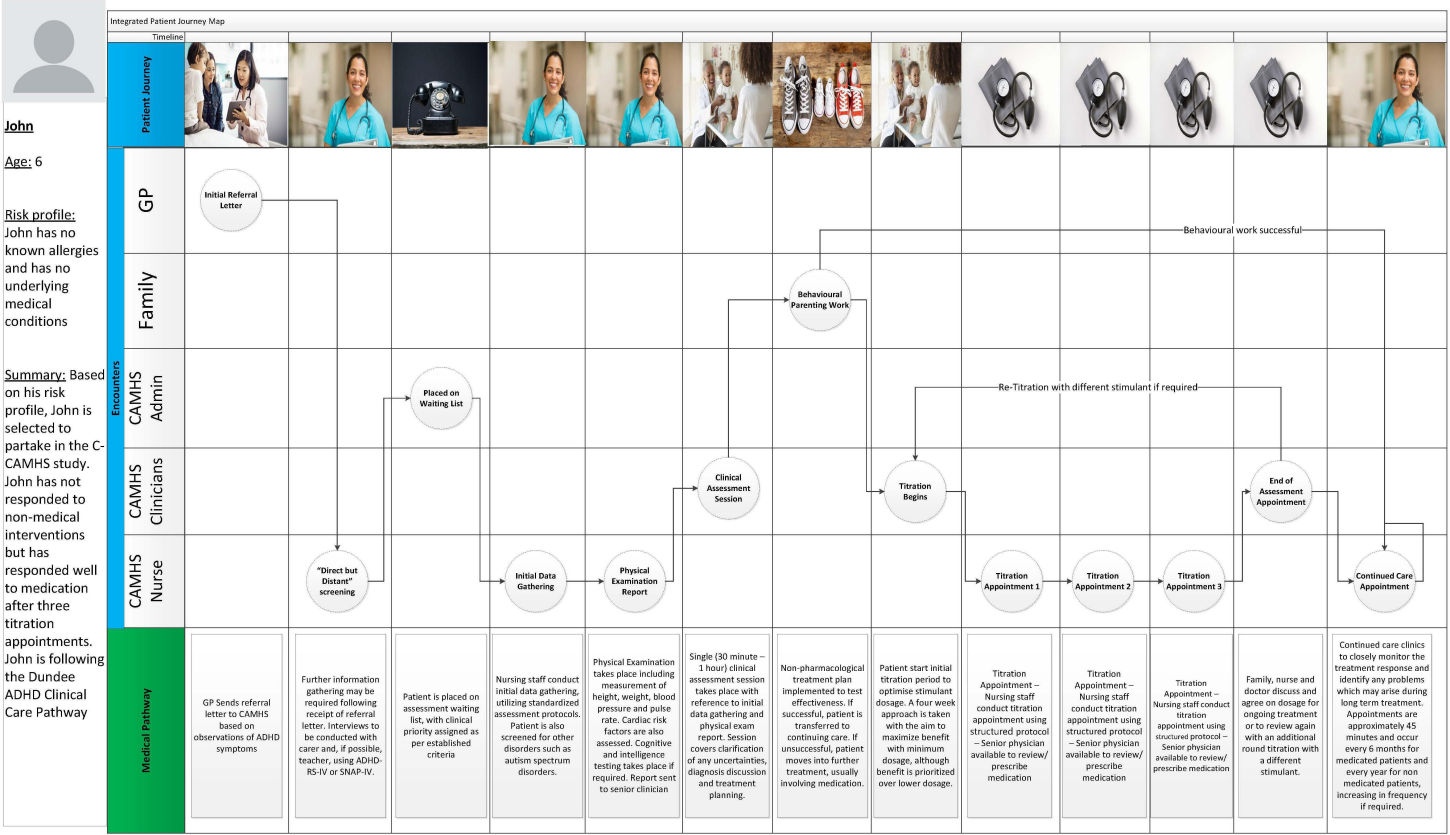
First iteration of the PJMA, visualizing the Dundee clinical care pathway as per interview and review of literature. Images under “patient journey” provided by Microsoft Office stock images, for illustrative purposes only. PJMA: patient journey map artifact.

In order to create the initial visualization, two unstructured interviews were conducted with a CAMHS clinician and nurse with experience in implementing the Dundee pathway. These interviews allowed the gathering of key data to assist in the development of the first iteration. Data collected in these interviews, along with a review of the literature, led to the creation of Figure 3, the first step in the process of developing a robust and verified PJMA.

The PJMA consists of a number of key areas:

- The left section of the pathway shows the patient persona, including the persona name, age, a brief risk profile, and a summary of the patient’s journey.
- The center section of the journey map highlights encounters that the patient will have with different actors in the pathway and each point along the journey represents an interaction.
- The section at the bottom highlights details for each interaction to provide greater context.

Each interaction leads to another, with two lines arising from “Behavioral Parenting work” to highlight that, if this intervention is successful, the patient may jump straight to the “Continuing Care Appointment” step. If unsuccessful, the pathway continues to titration [15]. The continuing care appointment represents a continuous cycle where periodic check-ins are conducted to ensure the patient remains happy with their treatment. A fork marks the “end of assessment appointment,” noting if the prescribed medication does not have the desired effect, additional rounds of titration are conducted with alternative medications.

Iteration one shows a single persona, but several personas were identified from interviews with CAMHS clinicians, with the persona in iteration one “John” representing a fictitious individual who would engage with every stage of the Dundee pathway. John has no known allergies and no underlying medical issues. John is therefore a suitable persona to illustrate the Dundee pathway. As John has no other medical concerns, it is highly likely that John can progress through the Dundee Clinical Care Pathway as described in the literature, without the need for additional interventions. John does not respond to behavioral techniques but does respond well to the first stimulant tested during titration.

#### Demonstration or Evaluation (Iteration One)

Demonstration of the first iteration of the PJMA, shown in Figure 3, was completed by presenting it to an expert of the original Dundee publication [15]. It offered an excellent opportunity to ensure the first iteration of the map was true to the original publication. Valuable feedback was received by the expert and feedback was generally positive.

Based on the feedback, it was decided that a second iteration was needed and that the feedback should be incorporated into it. General feedback highlights that the PJMA should be streamlined, pictures removed, and ad hoc appointments should be illustrated in subsequent iterations. Table 2 lists the interviewees for this iteration.

**Table 2.** Interview participant from iteration one by category.

Interview category	Number of interviews
Senior CAMHS^a^ clinician	1
CAMHS nurse	1
Dundee pathway expert	1

a CAMHS: Child and Adolescent Mental Health Service.

### Iteration Two

#### Design and Development (Iteration Two)

Creating the second iteration of the PJMA presented the opportunity to include multiple personas in the diagram by implementing unified modeling language (UML) principles to use the encounters as divergence points, or decision nodes, for each persona. Three personas are presented in the second iteration, representing three common patient scenarios, John remains as per iteration one, with Mary and Adam added as new personas.

- Mary differs from John as she has responded to nonpharmacological interventions and her parents agree that she does not require medication to manage her ADHD.
- Adam differs from John as he has not responded to nonpharmacological interventions and the initially prescribed stimulant has not had the desired effect, however, a second stimulant is effective.

To differentiate between each of the personas, color coding is used to represent each individual, with John represented in blue, Mary in pink, and Adam in green. Lines in black represent all three personas as the first number of encounters are the same for each persona.

Mary’s divergence point is during the “Behavioral work” encounter, as this was deemed effective for her, and she moved straight to “Continued Care.” Adam and John continued to titration where John was successful with his first stimulant. Adam required a second round of titration as the first stimulant did not have the desired outcome. This is illustrated by “Retitration.” All three personas finally reach “Continued Care,” which is illustrated by a circular arrow as this is a stage where patients are monitored continually until the age of 18 years.

At all relevant stages, an “as required” branch extends out into an “Other Problems Appointment” illustrating ad hoc appointments that may be necessary to discuss problems during treatment which may or may not be directly related to the treatment plan but may still be required for the patient’s well-being.

**Figure 4.**
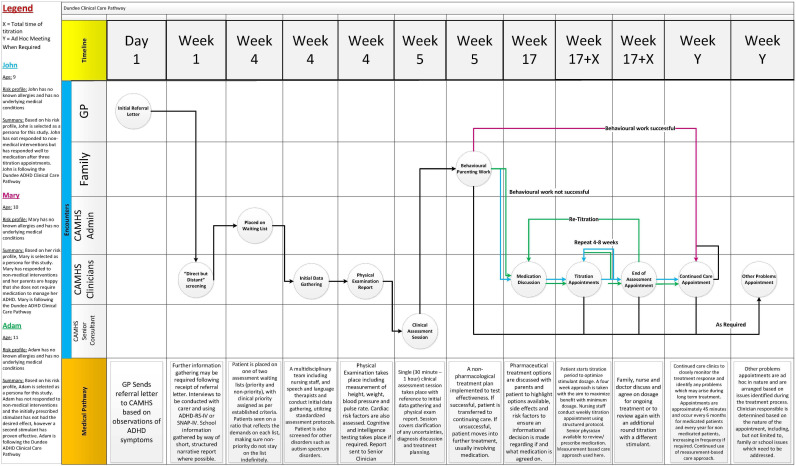
Second iteration of the PJMA, implementing feedback received from iteration one. Removal of images and ad hoc appointments added, among other changes. PJMA: patient journey map artifact.

For iteration two, images were removed from the diagram and replaced with a timeline section. This was identified during the semistructured interviews and represents a best-case scenario for the pathway. Two “wildcards” are included in the timeline. X represents the total number of weeks an individual is undergoing titration, which varies for each patient, and Y represents an unspecified as or when-required timeframe. Encounters have also been updated as per feedback from clinicians. Figure 4 highlights iteration two in detail.

#### Evaluation—Iteration Two

Evaluation of iteration two took place in two phases. Phase one was clinical verification to ensure the updated visualization was still an accurate representation of the pathway, with phase two involving four interviews of connected health consultants or academics to discuss the merits and drawbacks of the visualization for use as part of a requirements-gathering toolset. Table 3 lists the interviewees for this iteration.

**Table 3.** Interview participant from iteration two by category.

Interview category	Number of interviewees
CAMHS^a^ clinician	2
Social worker	1
Connected health researcher	2
Connected health consultant	2

a CAMHS: Child and Adolescent Mental Health Service.

Clinical accuracy validation of iteration two came in the form of interviews with two CAMHS clinicians, and a social worker, all of whom have experience working with or treating those with ADHD. Overall feedback on the diagram was very positive, with clinicians praising its accuracy and clarity. A key theme that was mentioned throughout the interviews was the multidisciplinary nature of CAMHS teams. Several clinicians, including nurses, consultants, occupational therapists, speech and language therapists, and social workers are involved in the screening and data-gathering process. It is important that these stakeholders are represented in the diagram to ensure their needs are met by future connected health apps.

The management of consent is another key concern. It is critical that informed consent is received at the point of referral to ensure that the CAMHS team is given permission to begin the screening process as this involves contacting multiple stakeholders in the patient’s life, including but not limited to, teachers, carers, parents, or any other relevant caregiver. This consent form will need to be well documented in any connected health app. A medication consent form will also need to be produced and signed by parents at the “Medication Discussion” stage as this protects all parties involved in the treatment process. This medication consent form needs to be captured in the diagram.

When discussing “behavioral parenting work,” it was noted that many parents do not decide to go down this treatment route as they may have already attempted this and concluded that this approach is not a suitable solution. As a result, parents have attended their general practitioner for a referral to CAMHS with the view of beginning pharmacological treatment. This is not, however, the case for all patients, and behavioral parenting work may still be a very effective solution when parents are provided with appropriate teaching materials and literature.

When discussing the “Other Problems Appointment” encounter, clinicians advised that this appointment generally occurs during treatment, either pharmacological or nonpharmacological but may occur at any stage of the pathway. Risk is the key driver of these appointments with the type and severity of the risk dictating who the patient is referred to and how quickly. The health and safety of the patient is always the key priority.

It was noted that, if “Behavioral Parenting Work” was successful, the patient is discharged from the services instead of being placed on a continued care plan. When discharged in this manner, patients can be referred to the service again, subject to a shorter waiting period.

It was noted that patients presenting to CAMHS often have comorbidities such as autism spectrum disorder (ASD), which may inhibit the effectiveness of stimulant medications.

Clinicians are also advised on the positioning of encounters based on their own clinical practice, with some screening or examinations taking place before or after others depending on the system in place within each specific service. It was, however, proposed that the relevant examinations would be identified within the diagram based on this feedback.

Following on from clinical verification of the diagram, the diagram was reviewed by four experts, consisting of connected health consultants and academics to provide feedback and help to identify possible areas for improvement. This yielded some very positive feedback, as well as very useful suggestions.

The colors for Adam and John’s personas were judged to be too hard to distinguish as the shades of blue and green were quite similar. The importance of keeping the visualization to a one-page size was emphasized, as a “one-pager” was deemed to be optimal from an information conveyance viewpoint. It was noted that some regulatory concerns pertaining to connected health apps were missing from the diagram. It was suggested that the map could be turned into a multilayered system, where additional information is overlaid, similar to “acetate sheets.” It was also noted that this iteration was quite clinically focused, and there was no mapping of the patient’s “emotional journey” through the service. The use of UML shapes was suggested to highlight where decisions are being made that may change the course of a patient’s journey. The benefits of visualizing the pathway to identify gaps where connected health can be embedded were emphasized. It was finally noted that images may be beneficial in visualizing who is responsible for each encounter.

### Iteration Three

#### Design and Development

Based on the feedback received from the evaluation of iteration two, it was decided that a third iteration of the visualization would be developed. This iteration, shown in Figure 1, has been updated to include the following changes.

The Adam persona has been updated to include a comorbid ASD diagnosis. This reflects the commonality of this comorbid diagnosis and the likelihood that ASD will impact stimulant performance. The colors of Adam and Mary have been switched to increase the contrast between John and Adam as both occupy similar locations on the diagram. A diamond shape is now used to illustrate where an individual patient’s journey may diverge based on clinical decision-making. A discharge encounter is added, and an “until 18” note is added to the continued care loop to illustrate that patients are discharged after effective behavioral work, or when patients become 18 years old. The medical pathway section was also updated to include relevant forms and the multidisciplinary nature of the data-gathering process. The legend was made more visible to highlight its importance. “Family” encounters were updated to “Caregivers” to include teachers, childminders, etc.

#### Demonstration or Evaluation (Iteration Three)

Demonstration of iteration three took the form of an email debrief containing Figure 1 to ensure that all feedback had been taken on board and that participants had the opportunity to provide final feedback. Overall feedback on the third iteration was very positive and participants highlighted their anticipation of the full publication of the work. This feedback gave the research team the confidence that the next step for this PJMA is to move on to the “Communication” phase of the design science methodology noted above and compile all iterations, feedback, and results into a research piece for consumption by the wider community. Table 4 notes the breakdown of individuals receiving a debrief email.

**Table 4.** Table of debriefed participants by category.

Participant category	Number of interviewees
Senior CAMHS^a^ clinician	1
CAMHS nurse	1
Dundee pathway expert	1
CAMHS clinician	2
Social worker	1
Connected health researcher	2
Connected health consultant	2
Total	10

a CAMHS: Child and Adolescent Mental Health Service.

## Discussion

### Main Findings

Feedback from clinicians and IS professionals suggests that using the IPJM mapping tool in Figure 1, effectively conveys the Dundee pathway to nonclinical audiences, enabling them to assimilate the information and subsequently facilitating more in-depth and focused conversations with clinicians when identifying pain points and opportunities for connected health adoption. This can then inform policy making in the health care area.

Based on the feedback from Clinicians and IS professionals, the modified IPJM diagram was well received by both groups. One may infer that visualizing the Dundee pathway is of benefit to stakeholders who are of a nonclinical background, as a visual aid to bring them up to speed on how the Dundee Clinical Care Pathway operates. The multidisciplinary nature of connected health apps requires input and contributions of individuals outside the clinical field, therefore, ensuring all team members working on a connected health project are aware of the standard care pathway. Figure 1 illustrates the Dundee pathway on an integrated patient journey map, the accuracy of which is verified by clinicians working in the field. This journey map may serve as a starting point for future research in connected health app design and development by effectively illustrating the Dundee pathway to nonclinical connected health team members.

As part of our research, we considered how the IPJM tool can be modified to enhance its accessibility. Using UML techniques, as reported by Curry et al [12], along with color coding, has allowed multiple personas to be layered onto one map, facilitating a more condensed, yet detailed, view of the pathway. UML also enables the presentation of clear focal points where clinical decisions divert individual persona’s trajectory on the pathway based on typical individual patient needs or circumstances.

Those interviewed noted that the one-page nature of the diagram is extremely powerful when conveying the details of the Dundee pathway. It reflects the full complexity of care to anyone working on a potential connected health design and implementation project. For instance, the single-page nature of the diagram supports robust conversations between clinicians and IS consultants to effectively identify possible areas for connected health adoption. One critical element of this is the added confidence it gives participants in terms of “sign of,” before substantial development begins. McCarthy et al [23] have identified the frequent changes in the path that occur during the development of IT solutions that address wicked problems in clinical care. These path changes are often disruptive and divisive for the development team as participants blame each other for misunderstanding the requirements. The robustness and ambiguity-removing effect of IPJM can secure a more reliable capture of requirements to support app development.

Figure 1 identifies clear areas where connected health can be effectively embedded. The “Discreet but Distant Screening” phase presents a clear opportunity to move from a paper-based approach to a software-assisted approach. The paper-based screening process takes a substantial amount of time, time which could be saved so clinicians can see more patients whilst a portal or an app does the computations of the scores automatically for them and provides full visibility on all cases in the waiting list, particularly the most acute ones.

During the “behavioral parenting work” phase, a connected health app could provide caregivers with relevant information in the form of text, audio, and video supports in sync with each relevant phase of the treatment. An app may also be interactive and collect feedback from caregivers and feed this information back to clinicians, who can see which techniques yield positive results and those that do not. This would allow clinicians to receive a clear and detailed picture of what is effective and subsequently allow them to make more informed decisions and provide more pertinent, faster advice.

The “Continued Care Appointments” phase presents an opportunity to use remote patient monitoring using Bluetooth-connected devices such as blood pressure monitors and connected scales. These devices may be connected to the app provided to each patient (eg, loaded on their phone or that of their caregiver) which will subsequently send the information to the clinician portal, eliminating the need for manual entry, and thus, eliminating the risk of human error when inputting results. Similar apps have been developed in the obstetrics area which showed great potential for the early detection of such conditions as preeclampsia [13,14].

**Figure 5.**
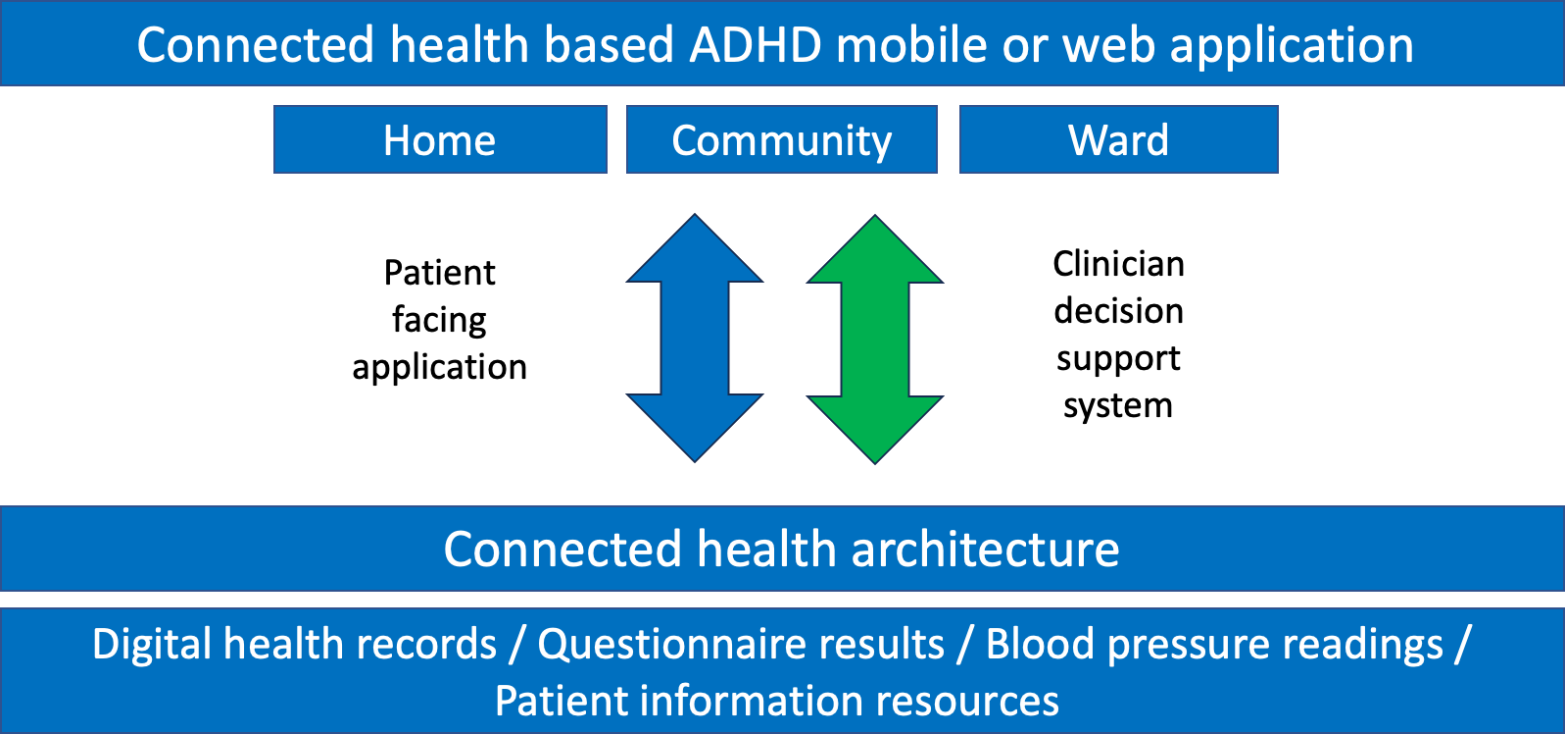
An architecture model for potential connected health apps in the ADHD area (adapted from Harris et al [24]). ADHD: attention-deficit/hyperactivity disorder.

Figure 5 proposes a generic architecture model for potential future connected health apps in the ADHD area, which abstracts and generalizes the above recommendations. The top layer of this diagram illustrates the interface layer used by patients and caregivers, either by way of a web portal or a mobile-connected health app. The second layer represents the operational areas for connected health apps, specifically within the home, in the community, or in a ward setting. The third level highlights two distinct touchpoints: the first being the patient-facing app, which gathers vital information from both home and community settings and feeds this data into the connected health architecture; the second touchpoint being the clinician support system, operating in community and ward environments, which assists medical professionals in making informed decisions based on data collected by and stored in the connected health architecture. Finally, the base layer in the model houses the model base (eg, algorithms) and database of the connected health app. This layer includes digital health records, questionnaire results, blood pressure readings, and patient information resources. Some rule-based engines and artificial intelligence components could also be located there to provide a higher grade of decision support to clinicians.

### Limitations and Future Research

The PJMA diagram is a clinical pathway representation, and as such, focuses on the pathway from the point of view of the clinician. There is certainly scope for future research focusing on the pathway from the patient’s perspective. Individuals who are using CAMHS services for ADHD treatment fall under multiple vulnerable categories as they are children or adolescents who are receiving mental health services. Substantial ethical concerns exist with interviewing such individuals, which is why we focused on the clinician and information technology side of the process. Our research was formative in nature, but further work in this domain from the point of view of the patient is required which will deliver additional insights into connected health apps for CAMHS.

### Conclusions

The multidisciplinary nature of connected health projects requires tools and techniques to be made available to allow nonclinical members of the team to effectively get acquainted with the clinical requirements of each particular project. Clinical journey mapping provides a way for team members to get acquainted with clinical practices, and also present their teammates with ways to identify key gaps where connected health systems can be embedded in clinical pathways in order to facilitate better use of clinical resources and ultimately deliver better patient outcomes.

Continued research in this area will help deliver continued progress in youth mental health, as well as health care in general, toward the well-being of future generations. Specifically, research into the design and implementation of connected health systems that can become embedded into the Dundee ADHD treatment pathway will be critical for the continued development of, and success of, Youth mental health services serving those with ADHD around the world. Research on software-based screening questionnaires, remote vital sign monitoring, and software-based teaching materials has the potential to support those with ADHD and their families.

## Supplementary material

10.2196/53617 Multimedia Appendix 1 Original ChatGPT Transcripts

## Abbreviations

ADHD: attention-deficit/hyperactivity disorder
ASD: autism spectrum disorder
CAMHS: Child and Adolescent Mental Health Services
IPJM: integrated patient journey mapping
IS: information systems
PJMA: patient journey map artifact
UML: unified modeling language
