# Supplementary material for: Clinician-Focused Connected Health Requirements Gathering for Attention-Deficit/Hyperactivity Disorder Through Clinical Journey Mapping: Design Science Study
Source: JMIR Form Res. 2025 May 26;9:e53617. doi: 10.2196/53617 (PMC12150888; doi:10.2196/53617)
Supplement: Multimedia Appendix 1 [file formative-v9-e53617-s001.docx]

**Multimedia Appendix 1**

Visualizing Dundee Clinical Pathway

This paper seeks to address three research questions. Firstly, how can the Dundee clinical care pathway be visualized using the integrated journey mapping tool? In response to this query, Figure 4.4 demonstrates the application of integrated journey mapping principles to represent the Dundee clinical care pathway. The diagram highlights each persona and employs color-coding based on the specific persona.

Secondly, how can the integration journey mapping tool be modified to enhance its accessibility? As indicated in the initial publication of the journey mapping tool, a primary concern was the substantial size of the original documents and their reliance on UML techniques. Consequently, the diagram has been condensed into an A4-sized format.

Lastly, does visualizing the Dundee clinical care pathway benefit stakeholders in identifying requirements for a connected health application designed to support the pathway's implementation? Feedback from clinicians and IIS professionals suggests that employing the updated clinical journey mapping tool, as illustrated in Figure 4.4, is advantageous in pinpointing crucial areas for integrating connected health within the service. The diagram effectively communicates the pathway to nonclinical audiences, facilitating their comprehension of its functioning and enabling them to propose areas for incorporating connected health within specific services. This ultimately leads to the creation of connected health solutions that aid in implementing the pathway.

This particular approach to clinical journey mapping may also be applied across various other services aiming to reach a discharge point as their ultimate goal.

Original Transcript

So this paper attempts to answer the three research questions noted above. One, how can the Dundee clinical care pathway be visualized on the integrated journey mapping tool? Based on this question, you can see that the Dundee clinical care pathway has been mapped using integrated journey mapping principles on figure 4.4, which highlights each persona on the diagram and color codes them based on the persona. Question two, how can the integration journey mapping tool be updated to improve its accessibility? So as mentioned in the original publication of the journey mapping tool, one of the key issues that was noted was that the original documents were quite large and utilizing UML techniques. This allows us to condense the diagram down into an A4 style sheet. And finally, is visualizing the Dundee clinical care pathway beneficial to stakeholders when identifying the requirements for connected health application designed to support the implementation of the pathway? Based on feedback from clinicians and IIS professionals, it is clear that utilizing the updated clinical journey mapping tool as highlighted in figure 4.4 is very beneficial when identifying key areas where connected health can be embedded within the service. As the diagram itself clearly conveys the pathway to a nonclinical audience, which then assists them in understanding how the pathway works, and ultimately will allow them to suggest areas where connected health can be embedded within the particular service to create connected health solutions to support the implementation of the pathway. This particular approach to clinical journey mapping can also be used across multiple other services, where the ultimate goal of the service or pathway is to reach a discharge point. Thank you.

Connected Health Overview

I am writing to provide an overview of the potential Connected Health application, as illustrated in Figure 4.1, which leverages the recommendations previously mentioned. The top layer of this diagram offers a glimpse at the web and mobile-based Connected Health application we are currently exploring.

The subsequent layer represents the operational areas for this application, specifically within the home, community, and ward settings. Delving deeper, the next level illustrates two distinct sections: the patient-facing application and the clinician support system. The patient-facing application gathers vital information from both home and community settings, while the clinician support system operates in community and ward environments to assist medical professionals in making informed decisions based on data collected by the patient-facing application.

The following layer highlights the Connected Health architecture, responsible for receiving information from the patient-facing application and distributing it to the clinical support system. Lastly, at the base of this illustration, you will find pertinent details associated with the Connected Health application. These include digital health records, questionnaire results, blood pressure readings, and patient information resources.

-------

Original Transcript

Figure 4.1 highlights the recommendations in an abstracted view of the potential Connected Health application which leverages the aforementioned recommendations. The initial top layer shows an overview of the potential Connected Health application we are looking at, both web and mobile based. The next layer represents the areas in which this application is currently going to be operationalised, in the home, in the community and in the ward. The next level shows the two discrete sections of the application, the patient facing application which takes place both in the home and the community setting in order to gather the appropriate information for the clinician support system. Which in itself then operates both in the community and in the ward setting, designed to assist clinicians in making clinical decisions by leveraging information connected by the aforementioned application. The next layer down is the Connected Health architecture which takes in the information from the connected patient facing application and disseminates it to the clinical support system. And finally the bottom layer shows the relevant information associated with the Connected Health application including digital health records, questionnaire results, blood pressure readings and patient information resources.
